# Development of an eHealth information resource for family carers supporting a person receiving palliative care on the island of Ireland

**DOI:** 10.1186/s12904-019-0457-y

**Published:** 2019-08-30

**Authors:** David Scott, Peter Hudson, Karen Charnley, Cathy Payne, Gareth Westcott

**Affiliations:** 1 School of Nursing and Midwifery, Medical Biology Centre, Queen’s University Belfast, Lisburn Road, Belfast, BT9 7BL UK; 2 All Ireland Institute of Hospice and Palliative Care, Our Lady’s Hospice and Care Services, Harold’s Cross, Dublin 6, Ireland

**Keywords:** Palliative home care, E-health, Care-givers, Education, Internet, E-learning

## Abstract

**Background:**

Many people receiving palliative care wish to die at home. Often, support from family or friends is key to ensuring that this wish is fulfilled. However, carers report feeling underprepared to undertake this role. This paper describes the process of developing a consensus and evidence based website to provide core information to help people support someone receiving palliative care on the island of Ireland.

**Methods:**

The project comprised three phases: (1) a review of systematic reviews facilitated the identification of core information needs; (2) content was developed in collaboration with a Virtual Reference Group (VRG) comprising patients, carers and professionals; and, (3) subject experts within the project team worked with a web developer to précis the agreed content and ensure it was in a format that was appropriate for a website. Members of the VRG were then invited to test and approve the website before it was made available to the general public.

**Results:**

Nineteen systematic reviews identified nine consensus areas of core information required by carers; a description of palliative care; prognosis and treatment of the condition; medication and pain management; personal care; specialist equipment; locally available support services; what to do in an emergency; nutrition; and, support for the carer. This information was shared with the VRG and used to develop website content.

**Conclusions:**

We engaged with service users and professionals to develop an evidence-based website addressing the agreed core information needs of non-professional carers who wish to provide palliative care to a friend or relative.

## Background

As populations’ age and increasing numbers of people are dying from chronic illnesses the demand for palliative care is growing [1, 2]. Most people who receive palliative care state that they would prefer to die at home [3, 4] and, as a consequence, providing home-based palliative care services is now a policy goal in many countries including Northern Ireland and the Republic of Ireland [4–7]. Moving palliative care services into the patient’s own home creates challenges for service providers. Evidence suggests that currently, there is a lack of research to guide policy makers and service planners and that the research evidence that is available is underutilised [8, 9]. A key issue is how to work effectively with family carers to ensure that they have the skills and confidence necessary to meet the patient’s needs [10, 11]. Previous research has demonstrated that often, carers find the transition from family member or friend to carer difficult, as they are required to re-define their relationship with their relative and assume often complex caring responsibilities [12]. Further research has illustrated that family carers can feel underprepared and question if they have the skills necessary to recognise and monitor symptoms, provide specialist technical care or, identify an appropriate time to contact a health professional [13–15]. This change in role and responsibilities is associated with problems among many carers including a deterioration in their overall health, sleeplessness, exhaustion, financial concerns and, anxiety or depression [11, 13].

The important role played by family carers in helping to manage chronic illnesses and providing palliative care is widely recognised and there is a need to support family carers by providing evidence based interventions and services that meet their needs [16]. Providing information that helps to empower and support family carers in a timely and efficient manner is a challenge for policy makers and service planners. Studies demonstrate that family carers benefit from the provision of core information to help them prepare for and respond to the role of supporting a family member as they approach the end of their life. However, the information that is currently offered to family carers can be variable and, in some instances, may not be offered at all [4, 17, 18]. Although the evidence base underpinning interventions that are delivered face to face to family carers is improving [17], these types of interventions can be resource intensive and may not be readily available to those living in remote or rural areas [16].

The use of eHealth platforms such as websites to provide information and guidance is becoming increasingly common in healthcare [19]. The proliferation and acceptability of the internet has provided an alternative format for sharing health-related information which is convenient, accessible and allows the user the opportunity to remain anonymous. The effectiveness of using a web-based approach to deliver health care interventions has been demonstrated elsewhere [20, 21] and has been recommended as a medium to help increase awareness and improve capacity regarding the provision of palliative care [22]. However, we should recognise that the use of websites to provide health related information may not be appropriate for all user groups particularly those who may not be regular internet users [20, 23].

The purpose of this project was to develop a consensus and evidence based website (that is, a website containing information that is supported by well-designed and well-conducted research). This website would provide core information to help guide and support family carers of palliative care patients on the island of Ireland and assist them in their caregiving role.

## Methods

### Development process

The study was facilitated through the implementation of a project team (*n* = 12) who provided project oversight. The project team were assembled based on relevant expertise which included: two palliative care consultants; palliative care nursing (PH); policy development (KC; CP); patient and carer advocacy; academic expertise (PH; CP; DS); and, website development (GW). The project comprised three distinct phases which are summarised here and outlined in the results section below. In phase one, a review of systematic reviews was undertaken to identify the research evidence relating to the core information needs of family carers providing palliative care. We also examined interventions identified in the reviews in order to ascertain and summarise the types of core information provided. In phase two, the project team used information gathered during the review to inform the development of draft core content and disseminated this information to a Virtual Reference Group (VRG) for comment, amendment and approval. The VRG comprised 25 individuals with a background in palliative care, service provision, service users, carers and advocacy groups from across the island of Ireland. VRG members were recruited from professional and voluntary organisations specifically to inform the development of this project and the membership was recruited to reflect the social portrait of communities in Ireland. VRG meetings were chaired by a member of the project team and VRG members could attend in person or could participate using video conferencing software. In phase three, the project team engaged with a web-site developer to transform the approved core information into content appropriate for a website. Again, the VRG were consulted during the development of the website and offered key insights that influenced its development.

## Results

### Phase 1: a review of reviews

This review aimed to identify, evaluate and synthesise the research evidence relating to the core information requirements of family carers needed to support them in caring for a relative or friend who was experiencing palliative care and consider, where possible, the core content provided and which strategies appeared most effective in delivering this information.

We undertook a review of systematically conducted literature reviews that explored the core information needs of family carers [24–26]. We initially established criteria for the inclusion and exclusion of studies and eligible reviews were those that examined the core information needs of family carers to help them prepare for and manage the role of caring for a relative or friend who required palliative care. Other inclusion and exclusion criteria included: only reviews that had been carried out since 2000 (we wished to capture current practice); reviews had to examine the information requirements of non-professional adult carers; review articles were included if they examined caring for someone with palliative care regardless of condition or treatment location; due to time constraints we examined only articles that were published in English. Further information is available on request from the corresponding author.

An information scientist assisted with the development of a search strategy designed to identify the largest number of potentially relevant studies (see Table 1). The search strategy was adapted to meet the indexing requirements of each of the electronic databases used in the review (Medline; PsychINFO; Cinahl; Web of Science; EMBASE; Social Policy and Practice; OVID journals and Scopus). In addition, keyword searches were completed on a range of internet search engines while the reference list of all retrieved articles were searched for potentially relevant review papers. Finally, professional and academic contacts of the study project team were contacted and asked to identify additional published reviews not identified by the review process. The searching process was completed in June 2016.

**Table 1:** Search terms employed

1. Palliative OR terminal OR terminally ill Or terminal care OR end of life OR Hospice OR death OR dying OR grief OR bereavement OR grief OR attitude to death	
2. Carer OR caregiver OR family caregiver OR spouse caregiver OR family member OR lay person OR consumer	
3. Adult	
4. Educate OR education OR instruct OR teach Or Coach Or tutor OR train OR support OR therapy OR interventions OR Care Or communication OR learn OR Information OR preparation OR role	

**Fig. 1:**
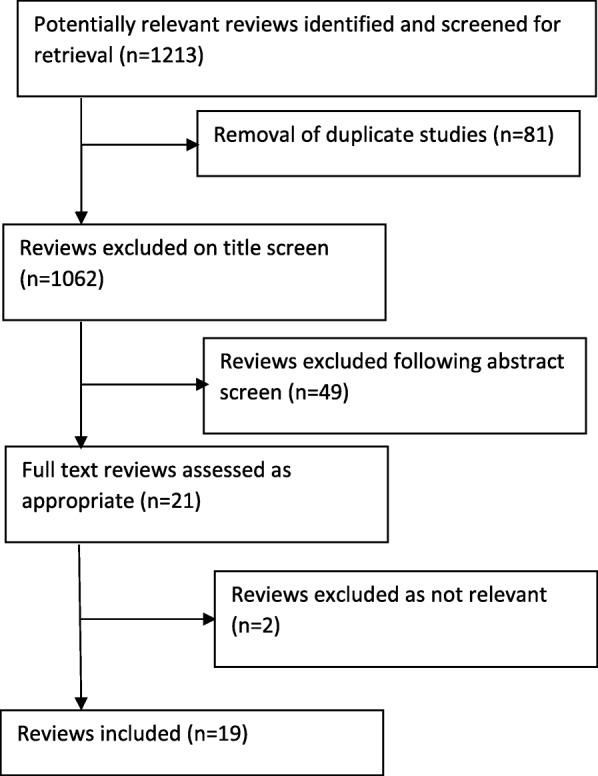
Flow of studies through the review

The searching process identified 1213 studies. We removed duplicate articles (*n* = 81) and read the retrieved titles in order to identify articles which clearly did not meet the inclusion criteria. This process facilitated the removal of papers which were not related to the subject or population of interest. Only studies with clearly irrelevant titles were removed (*n* = 1062) while ambiguous titles were retained for abstract screening. Abstract screening was undertaken on 70 papers using a ‘screening form’ which provided the basis of a systematic assessment of each paper according to the inclusion and exclusion criteria specified above. Following this process an additional 49 studies were removed. Remaining papers (*n* = 21) underwent data extraction by two research team members (DS, PH) using standardised pro-forma to ensure that the relevant information was systematically extracted from each eligible paper, during this process an additional two papers were excluded as they did not contain relevant information (see Fig. 1).

The methodological quality of the individual studies was assessed using a recognised evaluation framework [27]. Key information was tabulated to assist in the summary of findings and the synthesis of data. Data synthesis was completed by examining the tabulated data and by comparing and contrasting the findings to identify prominent themes (see Table 2).

**Table 2:** Review evidence examining the information requirements of family carers. (Published between 2000 and 2015)

Reference	Subject/population of interest	Total number of studies	Assessment of methodological quality	Analysis	Information requirements identified	Comments/conclusions
Capurro et al. (2014) [28]	Effectiveness of e-health interventions in meeting information needs of patients, caregivers and health professionals.	17 eligible studies.	No formal assessment of methodological quality is discussed.	Tabulated information on effectiveness of both qualitative and quantitative studies	Information on pain management, treatments, prognosis, reassurance on medication, symptom management, anxiety, death and, access to medical records.	Authors report use of e-health has been very limited and relatively few studies look at caregivers. Need for good quality RCTs.
Caress et al. (2009) [29]	Information & support needs of family carers of patients with COPD	35 eligible studies	No formal assessment of methodological quality is discussed.	Narrative review	No studies looked specifically at need. Four qualitative studies identified need for: more information on disease and more information on transition to becoming a carer (change to established roles)	Information needs of carers largely invisible. Carers need help in delivering physical and supportive care. Emotional support tasks may be greatest cause of burden.
Caress et al. (2009) [30]	Review of services to support family carers of patients with cancer	19 eligible studies	No formal assessment of methodological quality was undertaken.	Narrative review	Skills training provided to carers also symptom management, problem solving techniques, educational interventions to teach practical skills.	Studies show a wide variety in terms of the intervention and the diagnoses of patients. Interventions are likely to vary depending on stage of disease. Challenge to teach practical nursing skills to carers.
Docherty et al. (2008) [10]	Knowledge and information needs of informal caregivers in palliative care.	34 eligible studies	Yes, used a tool they claim has previously been used in studies of palliative patients/carers (Hawker et al., 2002).	Extracted data were tabulated and authors completed a narrative synthesis	Needs in relation to pain management, information on illness, prognosis, symptoms which may indicate disease progression, palliative care, spirituality, social welfare, psychological issues.	Major aim of palliative care is pain management and this is reflected in many studies – barriers include lack of knowledge, poor communication and variance in patient carer need. Info should be tailored to individual and disease stage.
Harding & Higginson (2003) [17]	Examine effectiveness of interventions to assist home-based carers of cancer and palliative care patients.	22 eligible studies	Yes, used a hierarchy of evidence approach (GRADE).	Extracted data were tabulated and authors completed a narrative analysis.	Home care, respite, social networks/activity groups, one-to-one interventions and, group work were all identified but – little information provided on information needs	Lack of evaluation studies in this area. Palliative carers often dislike leaving the patient to gain services for themselves. Need to prioritise evaluation studies.
Harding et al. (2012) [11]	How to support informal caregivers of cancer/palliative patients	33 eligible studies	Yes, JADAD score and GRADE	Considered meta-analysis but extracted data according to service design, tabulated outcomes and completed narrative review	Most studies are one-to-one interventions – findings generally positive. Group interventions are also generally positive	More evaluations are being conducted but evidence is weak – need to look at mechanisms of change in better defined models
Hinkle et al. (2015) [31]	Factors effecting family satisfaction with end-of-life care in the ICU	23 eligible studies	Yes, adapted CONSORT checklist	Extracted data were tabulated and authors completed a narrative analysis.	Few specific information requirements identified. Family support is key, support in decision making, knowledge of patient’s wishes about EoL care. Style of professional contact is important – empathetic, clear, early palliative care consultation is important.	Written communication may work well as carers may not retain verbal information at this time. Style of communication is important, empathy and explanations of process increase carer satisfaction.
Honea et al. (2008) [32]	Review to assess measures available to assess caregiver burden and interventions to supports oncology caregivers	20 studies examined interventions for carers	Yes, CASP	Extracted data were tabulated and authors completed a narrative analysis	Review found structured programs of information for carers – individual and group. Provide info on disease process, symptom management, psychosocial issues, resources, coordination of care, caregiver self-care	Study examines caregiver burden only. Key finding is the lack of interventions for caregiver strain and burden. Potentially valuable studies provide info on pain management, teach caregiver competence, discuss psychosocial issues, identify resources & coordinate existing care.
Hudson et al. 2010 [18]	Examine interventions that aimed to improve psychosocial support of family carers of palliative care patients	14 eligible studies	Yes, GRADE hierarchy of evidence scoring system	Extracted data were tabulated and authors completed a narrative analysis.	Some evidence that interventions aimed at psycho-educational, problem solving and cognitive restructuring may benefit. Some evidence that interventions should be targeted at specific problems experienced by a carer.	Some evidence that psycho-educational interventions may benefit but although there is more research it is still insufficient. Targeting interventions to individual need is likely to be benefice al
Jones et al. (2011) [33]	To assess the effectiveness of interventions that aim to improve the psychological & physical health of family carers	11 RCTs	Yes, used Cochrane’s guidelines.	Meta-analysis	Nine interventions provided direct support to the carer – weak evidence that these may reduce psychological distress in the short term and may improve QoL and coping skills – but no results were statistically significant. Indirect support to carers yielded no significant results.	There was evidence of the effectiveness of interventions directed at the carer but evidence based on a wide variety of studies and outcomes which makes pooling the data difficult. Some evidence that carers should be assessed and interventions should address individual need
McCorkle & Pasacreta (2001) [34]	Review and describe the needs of cancer caregivers during the palliative phase of the illness.	Unclear	No	Descriptive analysis	Carers are unprepared for role e.g. transport, emotional support, maintaining home, complex nursing tasks being undertaken by carers, stage of illness has impact on carers. Many studies show that carers find it difficult to access information. Carers have greatest informational need at; diagnosis, hospitalisation, start of new treatment and dying phase	There is relatively little research to guide interventions. Interventions may be about teaching carers nursing tasks to meet needs of patient. Some interventions develop individual and group interventions for carers. Little research evidence for either type of intervention.
Moore et al. (2013) [35]	The supportive & palliative care needs of patients with high-grade glioma & their carers	21	Yes, CASP tool	Extracted data were tabulated and authors completed a narrative analysis	Postoperative information to allow active involvement in care, disease and treatment information, side-effects of treatment, medication management, prognosis, financial information, navigating healthcare systems, information on resources such as support groups	Carers want consistent advice. Info needs change depending on illness trajectory Relevant/ timely information supports transition to caring role, raises awareness of services, helps address uncertainty and aids advocating role.
Morris et al. (2015) [36]	The needs of family carers supporting a relative who is dying at home.	28	No	Extracted data were tabulated and authors completed a narrative analysis	Information needs included pain relief, how to monitor and interpret symptoms, when to inform health professionals. Preparation for caring role – need nursing guidance at earlier stage Lack of practical support/poor communication is stressful	Carers reported both positive and negative experiences. Want to maintain normal life as long as possible Carer experience influenced by informal supports, professional help, ease of accessing technical expertise. Resources should be used to minimise burden.
Northouse et al. (2010) [37]	Review aims to analyse and assess the effectiveness of interventions provided to caregivers of cancer patients	29 Randomised Controlled Trials	None discussed	Meta-analysis	Majority of interventions included information on caring for the patient, maintaining family/marital relationships & carers caring for themselves. Meta-analysis of information only interventions (*n* = 3) were favourable with small to large effect size but these were small studies.	Majority of interventions were delivered to patients & carers jointly but great variety in content and duration of interventions. Meta-analysis showed that overall effect sizes were small to moderate but consistently associated with reduction in burden, improved ability to cope, increased self-efficacy and improved QoL.
Parker et al. (2007) [38]	Patient and carer end-of-life communication needs.	46	Yes, used a hierarchy of evidence system to identify methodologically weak studies	Narrative synthesis	Carers require more information on prognosis & death, particularly as illness progresses, options for treatment, survival timeframe, pain control, side effects of treatments, practical nursing skills to care for patient, how to deal with an emergency, fear of ding and spiritual issues.	Individual differences but carers require understanding so they can mentally prepare, organise lives and be a source of information to others. Style of discussion may be important, empathetic, who is present, sufficient time, acknowledged as an individual. As illness progresses patients may want less information while carers often wanted more.
Thompson et al. (2007) [39]	Do information and support interventions improve the QoL of people caring for a family member with dementia?	44 eligible studies	Yes, used Cochrane and CRD guidelines	Meta-analysis	Group-based interventions - found a significant improvements in depression for psycho-educational interventions. No significant differences for burden or supportive interventions. Individual-based interventions – improvements for depression, control group improved in measure of self-efficacy but neither were significant.	Trials are generally small, poor quality so difficult to draw conclusions. Small statistically significant improvements for group interventions on depression scores may not be clinically significant. Little evidence to support the universal effectiveness of interventions to improve lives of caregivers.
Ventura et al. (2014) [40]	Describe, evaluate & summarise literature relating to unmet need in home-based palliative care	15 eligible studies	Yes, Standard Quality Assessment Criteria for Evaluating Primary Research Papers (Kmet et al., 2004).	Extracted data were tabulated and authors completed a narrative analysis	Communication needs: carers require open & regular communication with professionals. Consistent message. Spiritual needs: fear of future, death is debilitating Practical needs: equipment, transport, household, finance. Information needs: Illness & prognosis, emergency response. Need to feel competent	Open communication with professional is major unmet need. Most needs identified were psycho-social or spiritual – physical needs appear to be met. Most included studies focus on cancer so may not be generalizable. Needs of patients and carers appear similar

This phase identified 19 eligible systematic reviews (see Table 2). Seven studies focused exclusively on patients with cancer, ten studies included carers of participants with a variety of conditions (including cancer), one study focused on Chronic Obstructive Pulmonary Disease and one on carers of patients with dementia. In terms of the ‘core’ information needs of carers, there was agreement about the types of information required which can be summarised under nine broad headings: a description of palliative care; information on the specific condition (prognosis and treatment); providing medication and pain management; providing personal care and hygiene; the use of specialist medical equipment; locally available professional supports; what to do in the event of an emergency; nutritional information; and, providing information to deliver psychological support and improve the carer’s confidence (see Table 3).

**Table 3:** Common core information needs of carers

Category of need	Examples of information and/or skills required
A description of palliative care	An explanation of palliative care, what it is, who it is for, what services are available and what to expect when caring for someone with palliative care needs?
Information on the condition, prognosis and treatment.	Specific information on the illness, recognition of common symptoms and their management. Likely prognosis. Symptoms and how to manage these such as fatigue, nausea, wound care, skin care, general care.
Medication and pain management	Knowledge of how to assess and manage pain; types of medication; dosage and possible side effects; giving injections / using syringe drivers.
Personal care/hygiene	General information on bathing, dressing, manual handling, continence/bowel management, fatigue, breathlessness.
Specialist equipment	The availability and use of specialist equipment such as hoists, oxygen, catheters etc.
Locally available professional supports	Information on locally available supports such as respite, sitting services, helplines, emergency contacts.
Emergency supports	Recognising and responding to an emergency, what to do, who to contact. How to communicate with healthcare professionals.
Nutrition	Dietary advice, feeding techniques, how to prevent dehydration.
Carer anxiety / confidence	Clarification about caring role and what is expected of you, reassurance around medication provision, frequently asked questions, decision making, communicating with your loved one, support materials (covering social, emotional, spiritual or physical care), emphasis on self-care, socialising when you are a carer, positive aspects of caring.

The reviews identified that the provision of information and training to family carers should be a priority as this would be likely to contribute to improved feelings of competence in undertaking caring tasks [10, 18, 30]. There was general consensus that a major contributing factor influencing a family member’s ability to undertake a caring role was a clear understanding of palliative care, what was expected of them as carers and, a clearly signposted pathway through which they could gain relevant knowledge and practical caring skills. There was some variation in the information needs of carers depending on their family member’s illness. For example, reviews which focussed upon cancer highlighted the need to provide training in specialist healthcare skills such as the administration of injections, the use of syringe drivers, medication dosage, side-effects and awareness of what to do in the event of adverse reactions to treatments [41, 36, 32, 38].

Several reviews highlighted that the information needs of family carers vary at different points during the course of the patient’s illness with carers requiring additional information as the patient approaches end-of-life including: what to expect, physical care, general coping, psychological care, information on available equipment and other resources such as hospice and/or respite care, how to deal with an emergency and, clear information on how to access professional help [14, 38, 40].

Due to their abridged nature, the systematic reviews provided little detail about the components of the specific interventions. The project team therefore examined a sample of ‘key’ original studies that were consistently cited in the reviews (*n* = 28) in order to gain a better understanding of the content of the individual interventions and how this information was presented to carers. This information was also collated and used to supplement the findings of the systematic review which was then shared with the Virtual Reference Group.

### Phase 2. Development of content

This part of the project sought to share the review findings with members of the Virtual Reference Group and explore their views on the content, design and features required of a carer’s website.

The review (supplemented by the descriptions of individual interventions) was summarised in a report which was provided to all members of the VRG. A series of meetings were convened in which participants could attend in person or virtually (using video conferencing software). In the first of these meetings participants discussed the review findings and provided feedback on the core areas of information identified and additional content that they felt would be particularly relevant to informal carers across different disease groups on the island of Ireland. To ensure clarity, these discussions were audio recorded, detailed notes were made on key topics and a list of action points were agreed with VRG members. These notes were used to ensure that the views of the VRG were reflected in the final core information provided in the website.

The meetings helped the project team to generate a first draft of content that would outline the core information required by carers. These topics included:

1. What is palliative care?
2. Caring for yourself.
3. Common illnesses that may require palliative care.
4. Caring for your relative at home.
5. Providing practical care.
6. Caring for your relative in hospice, hospital or residential home.
7. Advance care planning, legal matters and funerals.
8. Care as death approaches.
9. Bereavement.

Content development followed an iterative process where the project team initially composed written information based on these core topics and supplemented this information through the provision of links to existing web-based resources and content. This content was shared with members of the VRG who were invited to review and comment on the proposed information. VRG members provided feedback via email or telephone calls and this information was collated by the project team before a further meeting of the group as a whole was arranged to discuss progress. During this meeting participants were invited to reflect on the suggested content and provide comments. Discussions were focussed through the use of a topic guide. The topic guide required participants to read and reflect on the proposed content and consider: ‘Is the content relevant and meaningful?’; ‘Should there be additional content?’; ‘Is the content easy to read?’; ‘Is any of the proposed content unnecessary?’ Again, responses were audio-recorded and transcribed to ensure an accurate record of the conversation was available to guide content development. Following this meeting the content was reviewed by the project team and amended to reflect the consensus views of the VRG before again being shared with the group for a final time. The VRG was convened to ensure that the proposed content reflected the views of the group before progressing to website development. The development of the website did not begin until we had consensus agreement within the VRG.

### Phase 3. Website development

In this phase of the project we sought to translate core content into material appropriate for a website for carers on the island of Ireland. To consult with the VRG to ensure that the website reflected their priorities, views and experiences and also assess their views on the usability of the website.

The project team included a web design specialist (GW) employed by the All-Ireland Institute of Hospice and Palliative Care, the organisation that would host and maintain the final approved website within their current online portfolio. Subject experts within the project team undertook the task of précising the agreed content while ensuring that the information provided met the overall objectives of providing core information to assist family carers. In addition, an important component of this phase of the project was to undertake a scoping review of existing family carer related websites in order to avoid duplication and identify relevant existing web based resources that this site could link to. The subject experts within the project team identified and reviewed key national websites from UK, Canada and Australia (PH, KC). The sites were examined to ascertain if they appeared to be: evidence based; easy to understand; relevant to our identified core information domains and that their content was relevant to carers from the island of Ireland. The results of this exercise were summarised and shared with the VRG. A final online survey was used to ensure that the VRG could provide their views on the content, design and interactive features of the website and that the views of the group as a whole were reflected in the final product.

The web developer created a preliminary version of the website which contained links to other websites and/or resources. A meeting of the VRG was convened to discuss feedback from the online survey and to obtain face to face reflections on the draft website via exploring questions such as: ‘is the website visually appealing?’; ‘is the content easy to read?’; ‘is it easy to navigate your way around the website?’; ‘will the website be appropriate for use with a mobile device?’

Following this consultation, the web developer amended the visual appearance of the site and members of the VRG were again invited to use the site and comment on any issues. Google Analytics was then added to the website in order to monitor the number of users visiting the site and gain an understanding of how people were interacting with the site. The website http://www.carers.thepalliativehub.com/ was launched in May 2017 and by May 1st 2018 over 5, 500 people had visited the site with the chapter addressing ‘Caring for Yourself’ proving to be the most popular section. The site was designed so that carers could select individual areas or ‘chapters’ relevant to their needs or, if they desired, they could move through all the available content. Downloadable fact sheets and links to additional suitable website materials were also included for those who wished to learn more about specific topics of interest. The vast majority of traffic to the website is currently originating from direct visits, that is, people who type the address into the URL bar or follow a link from an unknown source. A campaign to improve the visibility of the website from search engines (Google searches, Bing searches and other search engines) is planned for autumn/winter 2018. The website will also have a content management system added, this will allow approved moderators who have little knowledge regarding website development to update the site. The process of maintaining and updating the website will be undertaken by the All-Ireland Institute of Hospice and Palliative Care.

## Discussion

National and international standards mandate that family carers of people requiring palliative care receive sufficient information to support them in their role [42–45]. Currently there is no systematic or consistent approach to the provision of information by health professionals to family carers. Consequently, the provision of information to family carers to help support them in their caring role can be inconsistent. One possible solution is through the use of information technology to provide appropriate information that is accessible to a wide audience of carers. While there are some good examples of e-health resources for family carers there is a lack of evidence based resources that comprehensively focus on the informational and support needs of family carers of people with advanced disease who require palliative care [16, 46, 47]. In addition, many people receiving palliative care want to be cared for at home but are unable to do so because their family carers lack the required information and support to assist them [10, 11]. This study utilised a ‘review of reviews’ in order to identify the ‘core’ information needs of family carers when providing palliative care to a relative. Having identified consistent core areas of concern; we entered into an iterative process of sharing information with a virtual reference group of professionals and carers, to determine if the identified core information needs were relevant and, to generate content to meet these needs. The consistent involvement of this stakeholder group was, we believe, an essential step in ensuring that the design and content of the website will be acceptable to carers of palliative patients. By providing high quality information to carers, including links to various support services and task specific training, it is envisaged that this resource will provide core information to help family carers assist their relative or friend to remain at home for as long as possible.

### Limitations

There are however several noteworthy limitations regarding this project. The availability of rigorous research is a requirement when providing evidence based care. However, despite the fact that the number of older people is increasing worldwide, there is well documented concerns about the lack of research available to guide the development of palliative care services [8, 48]. The development of this website was informed by a review of reviews and it was notable that there were relatively few review articles available. In addition, several of the included reviews discuss the quality of the original studies and highlighted that many of the evaluated studies were methodologically weak and, due to differences in the patient groups, interventions, outcome measures and study designs, it was difficult to meaningfully combine outcome data. It is therefore difficult to conclude that the review identified all of the ‘core’ information needs of carers. We sought to overcome this potential deficiency through consultation with professional colleagues and through extensive dialogue with a virtual reference group of professionals and carers. Although, given the circumstances, this was a rigorous approach; we accept that it is not a substitute for basing website content on evidence based findings. It is also worth noting that few of the reviews, or their contributing papers, provided information on the background of the carers who participated in the original studies. It is therefore difficult to be certain that the core needs identified in these studies would reflect the needs and views of all carer groups including those from minority communities such as; Lesbian, Gay, Bisexual, Transsexual and Intersex groups; culturally and linguistically diverse groups; or, rural and remote carers. Again, through targeted recruitment, the virtual reference group sought to be broadly representative of the current portrait of communities within Ireland. The contribution of this group was essential in ensuring that the views of minority groups were considered when developing the website content.

Furthermore, it should be noted that, the information contained in the website is designed to complement rather than replace the information provided directly to those caring for people at home by their treatment team. Indeed, successful uptake of the website will likely be contingent upon health professionals referring family carers to the site.

## Conclusions

The provision of palliative care is a priority for many countries around the world [4, 5, 10, 11] and the important role of informal carers in assisting family members receiving palliative care to remain at home is well recognised [49, 50]. Informal carers face the challenge of being both a recipient of and a provider of health care. This study sought to develop an evidence based website to deliver core information to family carers. Although there was a lack of rigorous research to clearly guide the development of this website the collaboration of academics, healthcare professionals service users and carers through a ‘virtual reference group’ was crucial in establishing a web-based resource which will serve carers across the island of Ireland and beyond. Through accessing the website it is hoped that carers will feel more prepared to manage the challenges associated with caring for a relative or friend receiving palliative care. Future research will examine the manner in which this site is utilised and through an ongoing programme of development the site will develop to meet the identified needs of users. This study also highlights that, in general, there is a need to develop research which will explore whether eHealth type interventions of this nature make a tangible difference to the wellbeing of family carers and the people they support.

## Data Availability

A copy of the review of reviews is available from the first author or from the All-Ireland Institute of Hospice and Palliative Care website.

## Abbreviation

VRG: Virtual Reference Group
